# Functional and evolutionary diversification of luciferase genes in *Metridia lucens* Boeck 1865

**DOI:** 10.1038/s41598-026-36319-2

**Published:** 2026-01-23

**Authors:** Luís B. Gabín-García, Carolina Bartolomé, Pablo Iglesias, Ánxela M. Estévez-Salguero, Laura Rodríguez de la Fuente, Xulio Maside, Jose A. Costoya

**Affiliations:** 1 Molecular Oncology Laboratory MOL, Departamento de Fisioloxia, CIMUS, Facultade de Medicina, Universidade de Santiago de Compostela, 15782 Santiago de Compostela, Galiza Spain; 2 Instituto de Investigación Sanitaria de Santiago (IDIS), 15782 Santiago de Compostela, Galiza Spain; 3 Grupo de Medicina Xenómica, CIMUS, Universidade de Santiago de Compostela, 15782 Santiago de Compostela, Galiza Spain; 4 Laboratório de Biologia Evolutiva, Faculdade de Biologia, Universidade de Santiago de Compostela, 15782 Santiago de Compostela, Galiza Spain

**Keywords:** Ecology, Ecology, Evolution, Genetics

## Abstract

Bioluminescent organisms have developed extraordinary adaptations to produce light using a luciferin-luciferase reaction, fulfilling various ecological functions such as predator evasion, prey attraction, and intraspecies communication. Although the earliest record in the marine environment dates back some 540 million years, the evolutionary origins of this phenomenon remain largely unknown in most species. In Metridinidae copepods, light production capability is facilitated by a luciferase gene duplication. This study focuses on characterizing the luciferase genes of *Metridia lucens*, a copepod widely distributed throughout global oceans, excluding the high Arctic. Despite being the first species described in this genus, the genomic sequences of its luciferase genes remained unknown prior to this investigation. Here, using an integrated approach combining molecular cloning and high-throughput sequencing, we isolated and characterized *M. lucens* luciferase genes. Our results revealed an unexpectedly high genetic diversity both within and between specimens, consistent with the presence of an extended gene family in this copepod’s genome. We identified three distinct luciferase gene lineages, each represented by several copies, expanding our knowledge of marine bioluminescence evolution.

**Supplementary Information:**

The online version contains supplementary material available at 10.1038/s41598-026-36319-2.

## Introduction

The ocean depths harbour a myriad of organisms, each with unique adaptations to dark and high-pressure environments. Among these creatures, bioluminescent organisms stand out for their ability to produce heatless light, a phenomenon that has been reported in hundreds of genera distributed over almost all phyla^1,2^. This natural light production is made possible by luciferases, a group of taxon-specific enzymes that catalyse the oxidation of light-emitting molecules (luciferins). The evolutionary origins and timings for the emergence of bioluminescence in most species are still largely unknown, although the earliest record in the marine environment dates back some 540 My^3^. The widespread occurrence of this phenomenon among marine species is associated with a wide diversity of functions such as evading predators, locating and attracting prey, and facilitating communication within species^1,4^. The importance of their role in the survival of these organisms is frequently preserved by the generation of gene families (groups of related genes sharing a common ancestor and performing similar or complementary functions) that arise through duplication events and allow these organisms to develop new functions and adapt to their environment^5^. An illustration of this is the duplication of luciferase genes within some copepods of the *Metridinidae* family^6^, which have evolved to optimize light production (reviewed in^7^). Bioluminescent reporters have been used in numerous applications, such as gene regulation and signalling, protein–protein interactions, drug screening, molecular imaging, cell-based assays, and non-invasive in vivo imaging (reviewed in^8^). The luciferases from copepods present several advantages for cell biology applications, including high secretability and ATP independence. They also show notably high enzymatic activity, that surpasses both Renilla and firefly luciferases under comparable experimental conditions^8,9^. Moreover, these enzymes exhibit remarkable stability under physiological conditions, including significant thermal stability, properties that have contributed to their expanding use as bioluminescent reporters in various research applications^7^. Researchers have explored pairing novel luciferases with modified substrates like coelenterazine-v (CTZ-v)^10,11^. Additionally, luciferase-fluorescent protein pairs employing Bioluminescence Resonance Energy Transfer (BRET) have been developed to shift emission toward the near-infrared spectrum, though this approach increases the overall protein size^8,12^. Coelenterazine analogues represent a promising strategy to amplify signal intensity, improve substrate bioavailability, and red-shift emission wavelengths. For example, soluble coelenterazine (CTZ-s) boosts signal output by up to 100-fold^13^.

Up to now, many copepod luciferase genes were cloned from different species^6,14–17^, and the luciferases from *Gaussia princeps* and *Metridia longa* have been used as bioluminescent reporters in various applications^18^. Additionally, our group has reported a biosensor based on *Metridia lucens* luciferase fused to an affibody to assess Her2 expression in living cells^19^.

*Metridia lucens* Boeck 1865 is a copepod that is common in the upper layers of all the oceans, except the high Arctic^20–23^. It was the first *Metridia* species to be described and it exhibits significant morphological differences from the more commonly studied species, *M. pacifica* Brodsky 1950. However, their precise taxonomic relationships are still a matter of debate^24^. Curiously, none of the luciferase genomic sequences of *M. lucens* have been cloned to date. By combining molecular cloning and high throughput sequencing, we characterized the genetic structure of the luciferase-coding loci in *M. lucens*. Our results reveal a complex genomic organization, suggesting the existence of an extended gene family of luciferase genes and indicating strong selective pressure acting on bioluminescence-related traits. These observations are discussed in the light of recent developments in this research area.

## Results and discussion

### Sanger sequencing

PCR amplicons from the *Luc1* and *Luc2* loci of *M. lucens* (*MlLuc1*, *MlLuc2*) varied in length from 1238 to 1268 bp, and from 894 to 1266 bp, respectively. Intron and exon boundaries of *MlLuc1* and *MlLuc2* sequences were determined using complete CDS and mRNA sequences from *M. pacifica* and *M. okhotensis* (AB371096.1, AB371097.1, AB674505.1, AB674506.1. AB195233.1, AB195234.1, AB674505.1, AB519699.1).

*MlLuc1* amplicons, as *Luc1* sequences from *M. pacifica* and *M. okhotensis*, encompassed six exons and five introns^6,16^. They exhibited high haplotype diversity, with 15 distinct haplotypes out of 16 cloned sequences (*Hd* = 0.99; Supplemental Table 1). Notably, a single individual (Ind. 1) displayed a different haplotype in each of the three clones analysed (Table 1). The observed variation mainly consisted of single-nucleotide substitutions, along with six indel variants, the largest being a 30 bp deletion in haplotype PCS-M1.5 (Supplemental Table 1). The high level of variation involved not only the introns but also the coding regions, which exhibited a considerable fraction of nonsynonymous mutations. The mean nucleotide diversity at nonsynonymous sites (π_A_) was 0.8%, one-twentieth the value of that estimated at silent sites (π_*S*_ = 16.0%; calculated using both synonymous and noncoding positions; Supplemental Table 2 ). This is consistent with existence of selective constraints on these sequences (purifying selection removes deleterious mutations). A scenario supported by the negative Tajima’s *D* values observed at *MlLuc1*, which suggest a significant deviation of the mutation frequency spectra sequences from the neutral expectations (Supplemental Table 2).

**Table 1:** Haplotypes detected by means of PCR, cloning and Sanger sequencing (PCS).

Target	Template	Clones sequenced	Haplotypes detected
*MlLuc1*	Pool 1	6	PCS-M1.1, PCS-M1.5, PCS-M1.6, PCS-M1.10, PCS-M1.11, PCS-M1.15
	Pool 2	5	PCS-M1.2, PCS-M1.3, PCS-M1.7, PCS-M1.9, PCS-M1.12
	Ind. 1	3	PCS-M1.1, PCS-M1.4, PCS-M1.8
	Ind. 2	1	PCS-M1.13
	Ind. 3	1	PCS-M1.14
*MlLuc2/MlLuc3*	Pool 1	5	PCS-M2.1, PCS-M2.3/ PCS-M3.4, PCS-M3.7, PCS-M3.11
	Pool 2	3	PCS-M3.12, PCS-M3.13, PCS-M3.14
	Ind. 1	2	PCS-M2.8/PCS-M3.15
	Ind. 2	4	PCS-M2.5, PCS-M2.6, PCS-M2.7/ PCS-M3.1
	Ind. 3	1	PCS-M2.9
	Ind. 4	2	PCS-M2.4/PCS-M3.1
	Ind. 5	8	PCS-M3.3, PCS-M3.5, PCS-M3.6, PCS-M3.7, PCS-M3.8, PCS-M3.9
	Ind. 6	4	PCS-M2.8/ PCS-M3.1
	Ind. 7	4	PCS-M2.2/ PCS-M3.1, PCS-M3.2, PCS-M3.10

Note: PCS-M1x, PCS-M2.x, PCS-M3.x, are the haplotypes detected for *MlLuc1*, *MlLuc2* and *MlLuc3*, respectively (see the results section).

**Fig. 1:**
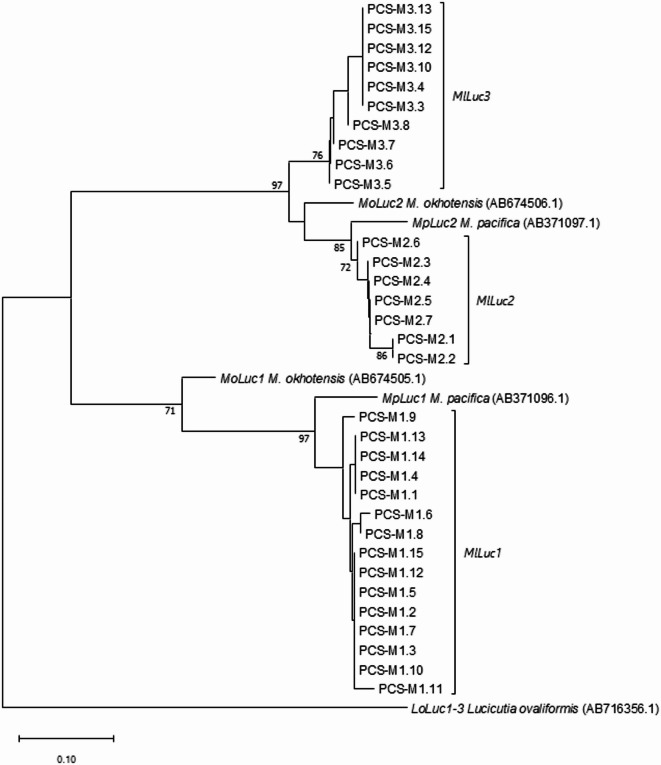
Phylogenetic reconstruction of the evolutionary relationships of the *Luc* haplotypes inferred using the Neighbor-Joining method. Bootstrap values (1000 replicates) higher than 70% are shown next to branches. The evolutionary distances were computed using the modified Nei-Gojobori method and are in the units of the number of synonymous substitutions per synonymous site.

Amplicons obtained with *MlLuc2* primers unveiled much larger genetic variation. A phylogenetic analysis revealed that the resulting haplotypes could be clustered into two well defined groups, hereinafter referred to as *MlLuc2* and *MlLuc3* (Fig. 1; Supplemental Table 3). *MlLuc2* and *MlLuc3* sequences encompassed five exons and four introns as previously described in *M. okhotensis*^6^. It should also be noted that *MlLuc3* haplotypes presented the two conserved tandem domains characteristic of luciferase genes (Supplemental Fig. 2)^6,25^. Also, in *MlLuc3* sequences the 5’ and 3’ boundaries of intron 1 were not conserved, and the lack of relevant *MlLuc3* mRNA data prevented the unambiguous determination of their true coordinates. However, potential alternative motives for both splicing signals can be found near their expected positions (two for the 5’-GT and three for the 3’-AG ends of the intron; at positions 101, 103, 170, 179 and 185, respectively; see the sequence alignment in the Supplemental data). Any of the six possible combinations of these alternative motifs would preserve the predicted open reading frames of the two adjacent exons and their coding potential. The N-terminal signal peptide would remain similar to that of *MlLuc2*, and the protein coding changes would affect the N-terminal variable domain, which is unimportant for luciferase activity^25^. At any rate, variation in intron–exon structure is not rare among *Luc* genes. For instance, the third intron of *Luc2* from *M. okhotensis* and *M. lucens* is missing in *M. pacifica*^16^.

**Fig. 2:**
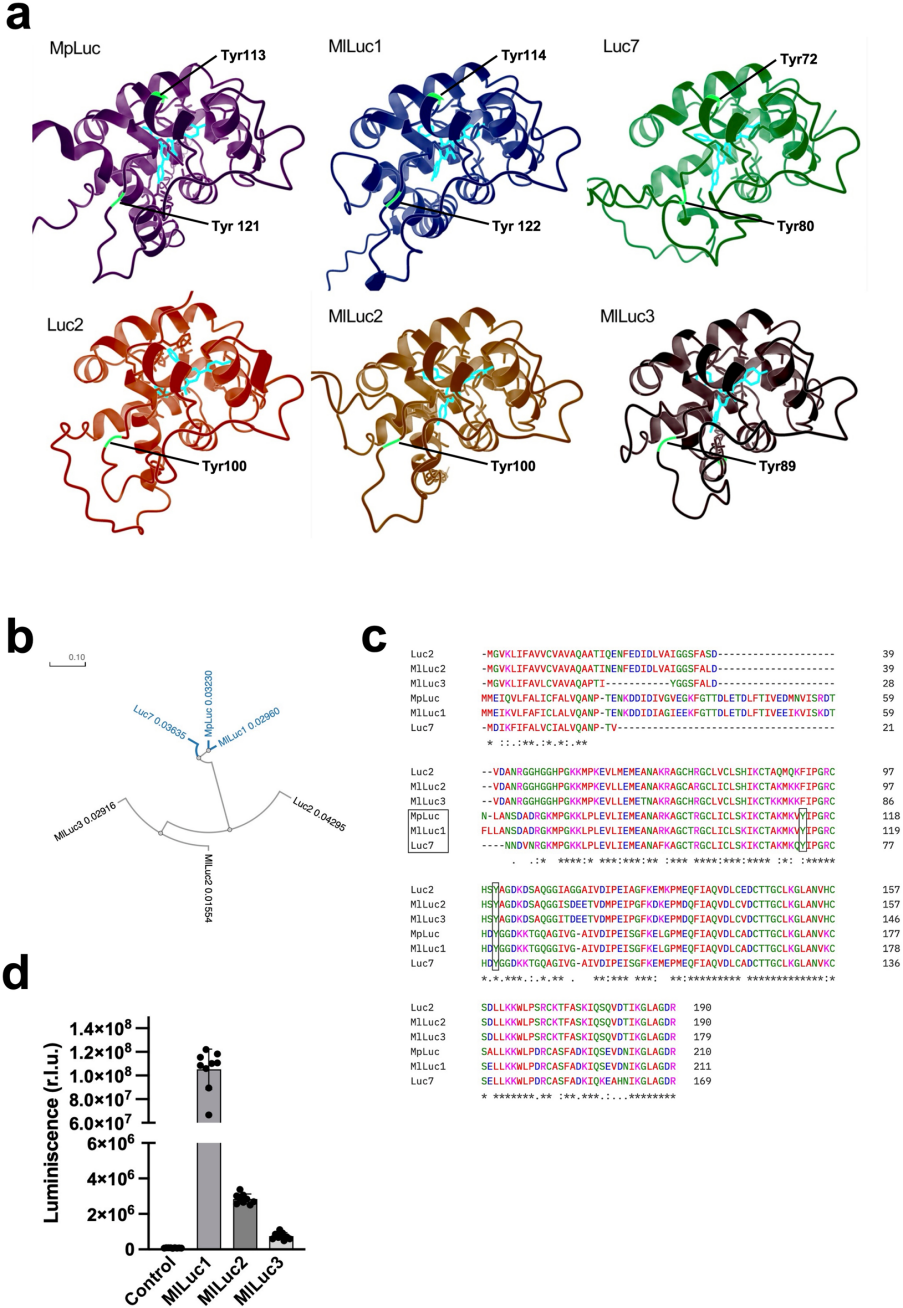
(**a**) Alphafold 3 model of the *Metridia pacifica* luciferase (MpLuc), *Metridia lucens* luciferase 1, 2 and 3 (MlLuc1, MlLuc2 and MlLuc3) and *Metridia longa* luciferase 2 and 7 (Luc2 and Luc7) bound to their substrate coelenterazine (in cyan). Tyrosine residues are labelled in green. (**b**) Phylogenetic analysis of Metridia luciferases sequences. The leaves of the tree represent individual proteins in the set, and their leading branches were colored according to the functions of the leaves, colored blue represents the luciferases that show both tyrosine residues in their sequence and colored black one tyrosine residue. (**c)** Sequence alignments of copepods luciferase proteins, highlighting within a black rectangle a region around Tyr 114 and 122 present in *Metridia lucens* luciferase 1 (MlLuc1), Tyr 113 and 121 in *Metridia pacifica* luciferase (MpLuc) and Tyr 72 and Tyr 80 in *Metridia longa* luciferase 7 (Luc7). The first Tyr is absent in *Metridia lucens* luciferase 2 and 3 (MlLuc2 and MlLuc3), and *Metridia longa* luciferase2 (Luc2). (**d**) Bioluminescence emission spectra of *Metridia lucens* luciferases (λmax = 480 nm). (**e**) Bioluminescent activity of MlLuc1, MlLuc2 and MlLuc3 at RT. Phylogram and sequences alignments analysis was performed using Clustal Omega Multiple sequence alignment using guide trees and HMM profile-profile techniques.

It must also be noted that not all the alleles could be clearly assigned to one or the other group since seven haplotypes (PCS-M2.8, PCS-M2.9, PCS-M3.1, PCS-M3.2, PCS-M3.9, PCS-M3.11 and PCS-M3.14; Supplemental Table 3) combined *MlLuc2* and *MlLuc3*-like stretches. These mosaic sequences could either correspond to true recombinants or to experimental artefacts occurred during the PCR. Several lines of evidence suggest that at least some of them are true alleles: (i) some of these haplotypes were found in different individuals (e.g. PCS-M2.8, PCS-M3.1; Supplemental Table 3), (ii) PCS-M2.8 was detected in PCS and in MPS data, which originated from independent PCR reactions (see the Materials and Methods and the Massive Parallel Sequencing sections), and (iii) PCS-M3.1, PCS-M3.2 and PCS-M3.9 share three exclusive nucleotide variants in their *MlLuc2*-like segments (pos. 1139, 1162 and 1163), which were not found in any of the other *MlLuc2* alleles detected in this study. Indeed, this observation suggests that these three alleles originated in a single recombination event in a distant past and now segregate in the *M. lucens* population and have accumulated some nucleotide divergence. At any rate, the recombinant haplotypes were removed from all subsequent evolutionary analyses to avoid interference in the inferences due to the different evolutionary histories of these segments.

*MlLuc2* and *MlLuc3* sequences displayed extensive haplotype variation (*Hd* = 0.91 and 0.96, respectively). A BLAST analysis of the *M. lucens* transcriptome (SRA: SRS3136877)^17^ allowed the identification of numerous *MlLuc2* haplotypes, many of which displayed intron retention, suggesting high levels of alternative splicing at this locus. In contrast, no *MlLuc3* sequences were found in the transcriptome, highlighting the role of alternative transcript processing in generating diverse luciferase-derived products across individuals.

The average nucleotide diversity of *MlLuc2* sequences was 15.6% at silent and 0.1% at nonsynonymous sites, respectively (Supplemental Table 2). Two of the six *MlLuc2* haplotypes, PCS-*MlLuc2*.1 and PCS-*MlLuc2*.2, displayed a 182 bp insertion that spanned a large fraction of intron 2 (Supplemental Table 3). Most single-individual samples had just one *MlLuc2* allele, except for Ind. 2 which had three (Table 1 and Supplemental Table 3).

*MlLuc3* haplotypes displayed significant length polymorphism, and all of them were shorter than *MlLuc2*, due to the occurrence of several deletions (Supplemental Table 3). The mean nucleotide diversity (π) at silent and nonsynonymous sites was 13.0% and 0.7%, respectively (Supplemental Table 2). Of note is that six *MlLuc3* haplotypes shared a large 80 bp deletion in exon 4 that involves part of the second conserved functional domain (Supplemental Table 3 and Supplemental Fig. 2). It is also worth noting that Ind. 5 and Ind. 7 harboured six and three distinct *MlLuc3* alleles, which suggests that in these specimens these sequences are coded by at least three and two different loci, respectively.

### Massive parallel sequencing

To further explore the high levels of allele variation observed within and among individuals, and the presence of three types of sequences (*MlLuc1*, *MlLuc2* and *MlLuc3*), a shorter fragment of the luciferase genes (~ 360 bp for *MlLuc1* and *MlLuc2*, and ~ 300 bp for *MlLuc3*) was deep sequenced in three single-individual samples (Ind. 8, 9 and 10). Seventy-eight thousand and six reads were obtained using the Ion Torrent PGM technology (21,472, 27,189 and 29,345 from Ind. 8, Ind. 9, and Ind. 10, respectively), 65.4% of which were longer than 150 bp (51,047 reads).

#### Luciferase MlLuc1

Ion PGM produced 3,820 *MlLuc1* reads > 150 bp which, after *Amplian* filtering, yielded ~ 1,257 *MlLuc1* reads with a posterior probability greater than 0.95. These underwent manual inspection and a stringent filtering process (see the methods section), resulting in the identification of six true haplotype candidates (MPS-M1.1, MPS-M1.8, MPS-M1.9, MPS-M1.14, MPS-M1.22 and MPS-M1.25; Supplemental Table 4). Ind. 8, Ind. 9, and Ind. 10 presented four, two and six distinct haplotypes each, respectively, all of which were detected in two or more independent samples.

There was substantial variation in the sequence read counts representing each haplotype. For instance, M1.1 and M1.25 represented 76.1% and 12.5% of the total number of reads of the selected haplotypes, respectively (Supplemental Table 4). This variation was also observed across samples, although read counts did not correlate with the haplotype diversity observed across individuals. For instance, although Ind. 9 had the highest number of filtered reads (*N* = 422), it presented just two candidate haplotypes, whereas Ind. 8 and Ind. 10, with 299 and 279 reads each, exhibited four and six haplotypes, respectively. These patterns of variation likely reflected the effects of a combination of factors such as haplotype diversity, copy number variation, and differential amplification rates across haplotypes.

Despite exhaustive filtering, to discriminate sequencing errors from genuine mutations is a major challenge when MPS platforms are used. To further validate the authenticity of these haplotypes, the *MlLuc1* amplicon libraries used for MPS of Ind. 8 and Ind. 10 were cloned and Sanger sequenced (12 and 22 clones, respectively). Nineteen different haplotypes were obtained this way, four of which had also been previously detected by PCS or MPS: M1.1, M1.8, M1.22 and M1.25 (Supplemental Table 4). There was a positive correlation between the number clones and the read counts across haplotypes (*R*^2^ = 0.77), which probably reflected variation in the representation of the different haplotypes in the amplicon libraries. The lower number of haplotypes identified by MPS as compared with PCS suggests that the filtering protocol of the MPS data was very stringent, perhaps even to the point that some potentially true haplotypes might have been discarded. Indeed, fifteen haplotypes obtained by PCS were not detected by MPS, which means that they were probably present at low frequencies in the *MlLuc1* libraries. To ascertain whether the haplotypes with less supporting evidence (e.g., those present in a single clone) were true haplotypes or could correspond to experimental artefacts (i.e., incorporation errors during PCR), their mutation profiles were compared with those of the haplotypes with more support (i.e., detected in two or more independent samples). The mean number of nucleotide changes that separated the former from their closest highly supported haplotypes was nearly identical to that observed among the latter (2.4 ±0.30 vs 2.2 ± 0,48; mean ± SE), indicating that haplotypes with weaker support did not present fewer mutations than the rest as would have been expected if they were sequencing errors. This evidence is in line with the hypothesis that they represent true alleles.

Overall, the MPS and PCS data are consistent with high levels of within-individual diversity of *MlLuc1* alleles. Ind. 10 presented 18 different alleles, which implies that there must be several copies of this locus in the *M. lucens* genome. This also holds true for the other two specimens, Ind. 8 and 9, which harbour seven and two haplotypes each (Supplemental Table 4). Even when only alleles supported by more than one type of evidence were considered (e.g., alleles found in more than one sample or detected by MPS and PCS), the data suggest the presence of four *MlLuc1* alleles in Ind. 8 and six in Ind. 10. It should also be noted that given that the MPS analysis covered only about one fourth of the full-length *MlLuc1* sequence, the total number of distinct *MlLuc1* alleles per individual was likely underestimated, as illustrated by the fact that a single MPS haplotype (MPS-M1.1; Supplemental Table 4) shared sequence with three PCS haplotypes, PCS-M1.1, PCS-M1.2 and PCS-M1.4 (Supplemental Table 1).

#### Luciferase MlLuc2

MPS of the three samples (Ind. 8–10) yielded 2,539 *MlLuc2* reads, including 1,161 reads with a posterior probability greater than 0.95. Subsequent filtering, together with PCS data, defined 12 potential true haplotypes (Supplemental Table 5). However, the extensive haplotype diversity observed among the *MlLuc2* clones (*Hd* = 0.91) suggests that some genuine MPS alleles might have been excluded due to the stringent filtering criteria applied. For example, two haplotypes sharing three unique nucleotide changes were omitted because they were found in single individuals, where they represented less than 15% of the reads (data not shown). Given the low probability of these three shared variants arising independently from sequencing errors in two different samples, it is likely that these alleles were genuine despite their exclusion. As in *MlLuc1*, the finding of more than two *MlLuc2* alleles in single individuals, both by PCS and MPS (Supplemental Table 5), suggests the existence of several paralogous copies of this luciferase gene in the *M. lucens* genome.

#### Luciferase MlLuc3

MPS produced 4,325 *MlLuc3* reads > 150 bp, 3,167 of which had a posterior probability greater than 0.95. Once filtered and compared with the sequences obtained by PCS, they resulted in 11 true haplotype candidates. As described for *MlLuc1* and *MlLuc2*, some *MlLuc3* PCS haplotypes were identical in the MPS region (Supplemental Table 6), meaning that the analysis of this MPS shorter fragment captured only a fraction of the total *MlLuc3* allele diversity.

The most frequent haplotype was MPS-M3.1, which was present in eight out the twelve samples analysed and represented between 58 and 93% of the reads detected in the three individuals analysed by MPS (Supplemental Table 6). Three other haplotypes were detected in more than one specimen: MPS-3.4 was only marginal in Ind.8 and Ind.9 but represented 31% of the reads from Ind.10; MPS-3.7 displayed only three reads in Ind.10, but was present in one of the pools (Pool1) and in Ind. 5, and MPS-3.8 was detected in the three specimens analysed by MPS at low frequencies (4–10% of reads), yet the two mutations that define this allele were also detected in a different haplotype (MPS-3.9).

### Evolutionary analyses

The phylogenetic reconstruction of the evolutionary relationships of the *Luc* genes allowed for some inferences into their origin in the genus *Metridia* (Fig. 1): (i) the deep separation of the *Luc1* and *Luc2* branches suggests that these genes originated from a duplication prior to the speciation events that generated the three species included in this analysis: *M. lucens*, *M. pacifica* and *M. okhothensis*. Indeed, the mean synonymous divergence (*K*_S_) between the *Luc1* and *Luc2* gene families in the three species (82.0%) is about four times larger than the mean synonymous divergence across the three species: 21% and 15% for *Luc1* and *Luc2*, respectively (from data in Supplemental Table 7). (ii) *M. okhotensis* diverged from the common ancestor of *M. pacifica* and *M. lucens*. The mean *K*_S_ between *M. okhotensis* and *M. pacifica* or *M. lucens* is twice as large as that between the two later species (*K*_S_ = 22.2% vs 9.7%). And (iii) *MlLuc3* sequences likely originated from a duplication of *Luc2* that predates the split between *M. lucens* and *M. pacifica* (mean *K*_S_ between *MlLuc2* and *MlLuc3* = 16.4%).

Nonsynonymous sites at the *Luc1*, *Luc2* and *Luc3* genes evolved at a much lower rate than synonymous sites across all taxa. Mean estimates of nonsynonymous divergence (*K*_A_) were 2.2% for *Luc1* and 3.1% for *Luc2* and *Luc3* (Supplemental Table 7; interspecific comparisons for *MlLuc3* sequences were done using *Luc2* from *M. pacifica* and *M. okhotensis*; see the methods section). Indeed, the average *K*_A_/*K*_S_ ratios were much smaller than 1 for the three genes, which is consistent with the action of purifying selection purging deleterious nonsynonymous mutations from the population and thus maintaining their functionality over time. This evolutionary scenario was further supported by the results of the McDonald and Kreitman test, which compares the ratios of fixed and polymorphic changes at silent and nonsynonymous sites. Under neutrality, these ratios should be the same. *MlLuc1* and *MlLuc3* departed from neutral expectations and displayed a statistically significant dearth of fixed nonsynonymous changes, an expected outcome of purifying selection (Supplemental Table 8). Contrastingly, *MlLuc2* displayed an inverse pattern, with a slight, although non-significant, excess of amino acid-replacement substitutions. Indeed, the ratio of fixed to polymorphic non-synonymous changes for this family is 8.5:1, as compared with 1:3.6 and 1.1:1 for *MlLuc1* and *MlLuc3*, respectively. These results could be interpreted as evidence that although the *Luc* genes in *M. lucens* are now under the main effect of purifying selection to preserve their functionality, they likely experienced distinct evolutionary forces that favoured their divergence. This is in good agreement with recent evidence that *Metridia pacifica* Luc1 and Luc2 luciferases have distinct enzymatic properties: *MpLuc1 *has been associated to sharp light emissions, whereas *MpLuc2* to slow build-up but much longer lasting luminescence events^16^. These different properties might be used for different purposes, such as startling predators or counter illumination^4^. In this context, it could be speculated that the third luciferase in *M. lucens* has a, yet uncharacterized, specific functional property that conferred a selective advantage to its bearers. Interestingly, the three *Luc* gene families present some fixed amino acid changes in their two tandem functional domains, although their characteristic five *Cys* residues remain intact (Supplemental Fig. 2)^25,26^. Overall, this whole scenario would be consistent with a model of gene duplication followed by functional divergence and reinforcement (reviewed in^27^). That is, (i) the duplication of an ancestral *Metridia* luciferase gene originated two paralogous copies that develop distinct functional capabilities that can be found in all *Metridia* species (*MlLuc1* and *MlLuc2*)^6^. (ii) In the ancestor of *M. lucens* the duplication of *MlLuc2* generated a third paralog copy (*MlLuc3*). (iii) Further duplication events produced an undetermined number of new copies of these three master genes that retained and reinforced the function of its parental paralogs, giving rise to three distinct luciferase gene families. The fact that nucleotide divergence is much larger among than within gene families suggests that the reinforcing duplications occurred after functional divergence. Overall, this represents an exceptional organization of the luciferase genes among copepods, where they are generally present in one or two isoforms; albeit *Pleuromamma xiphias* and* Metridia longa* with three and four isoforms each^7^. At any rate, the number of copies of each gene type remains unknown in most species.

### Structure and function analysis of luciferases of *Metridia lucens*

It has been reported that the residues Tyr72 and Tyr80 are key for the formation of the active site of the luciferase of *Metridia longa* (Luc7). Tyrosine substitutions do not eliminate their enzymatic activity although significantly reduce relative specific activity and change bioluminescence kinetics. Additionally, the tyrosine replacements have no effect on bioluminescence spectrum. Interestingly, the substitution of Tyr72 to Phe present in Luc2 isoform or in the Luc7 Tyr82 mutant of *M. longa* decreases the intensity of luciferase intrinsic bioluminescence activity, and this diminution seems that is dependent of the temperature. The optimum temperature for Luc7, as well as for Y80F mutant, is at 12 °C, whereas the substitution of Tyr72 shifts it for 5 °C toward low temperature^28^. An AF model analysis of the sequence alignments of Luc7, Luc2, MpLuc, MlLuc1, MlLuc2 and MlLuc3, revealed that Luc7, MpLuc and MlLuc1 conserve this Tyr, while in MLuc2, MlLuc2 and MlLuc3 is substituted by a Phe (Fig. 2a and Fig. 2c). To identify possible convergent evolution of luciferases functionality, we built a phylogenetic tree of these Metridia luciferases (Fig. 2b). We demonstrate that, although the three types of luciferases of *Metridia lucens* show the same bioluminescence spectra, MlLuc1 shows higher bioluminescence intensity than MlLuc2 and MlLuc3 at RT, as it was also described for Luc7 and Luc2/Luc7 Y72 mutant (Fig. 2d and e). Intriguingly, the global geographic distribution patterns of these Metridia species are quite broad, implying that these species can cope with different water temperatures. The existence of diverse luciferase isoforms may provide a potential strategy for these marine species to adapt to the different thermal conditions and spread their spatial distributions.

## Conclusions

Overall, the combined sequencing strategies from individual and pooled specimens support the existence of three distinct potentially active luciferase-like sequence families in *M. lucens*: *MlLuc1, MlLuc2 and MlLuc3*. The latter is a novel family that originated from duplication of *MlLuc2* in the common ancestor of *M. lucens* and *M. pacifica*. These gene families evolved under the predominant effect of purifying selection, and fixed sequence variants in the two functional domains would be compatible with functional divergence.

## Material and methods

### Collection of the copepods

Zooplankton samples were collected from the Irish Sea. Following collection, copepods were fixed in 70% ethanol. Fixed copepods were examined under a stereomicroscope and *M. lucens* copepods were sorted (ca. 50 individuals) for further analysis (Supplementary Fig. 1). Morphological classification was confirmed by amplification of conserved 18S sequences (M18S-F: 5' CTTTGAGCTGATCGCATGGC 3’, M18S-R: 5' AGTAAACCTGCCAGCATCCC 3’).

### PCR-cloning and Sanger sequencing

Genomic DNA was extracted from two pools of 5–10 individuals and from single *M. lucens* specimens (individuals 1–7; Table 1). The coding sequence for *MlLuc1* was amplified using *MlLuc1* primers, which span positions 34–1145 of the *Luc1* gene from *M. pacifica* (MpLuc1; GenBank Accession Number: AB371096.1). Primer pairs for *MlLuc1* were M1-F: 5' AACTGGATCCAAAAGGAAA 3’, M1-R: 5' ATGAGKYAAGCATATCATGATC 3’, M1-F2: 5' GGAGACAACTGGATCCAAAAGG 3’, M1-R2: 5' GCATATCATGATCCAG TTATC 3’, M1-R2b: 5' TCTCTTGTTCTGTTCTGTCAGGT 3’. Those used for *MlLuc2* encompassed the positions 28–1069 of MpLuc2 (GenBank Accession Number: AB371097.1). Primer pairs for *MlLuc2* were M2-F: 5'-TCCAAACYGAAAGGTACTC-3', M2-R: 5'-AAGTATCATCATCAAATTATCCA-3', M2-F2: 5'-GAGTCCAAACTGAAAGGTACTCA-3', M2-R2: 5'- GCCATTTTTAACATCATTGGGCT-3'. The resulting products were cloned into pGEM-T vectors, and plasmids were Sanger-sequenced using T7 (forward) and SP6 (reverse) primers.

Sequences were checked for accurate base calling using Codon Code Aligner (CodonCode Corporation) and manually aligned with Bioedit^29^.

### Massive parallel sequencing (MPS)

#### PCR-amplification

DNA individually extracted from three further specimens (individuals 8–10) was amplified for subsequent Ion Personal Genome Machine sequencing (Ion PGM, Life Technologies). Primer pairs for luciferases *MlLuc1* and *MlLuc2*/*MlLuc3* were MF1: 5'-ATGATGGAAATAAAAGTTCTTTTTGC-3’ and M1R-seq: 5'-GAAGATTAAAATCCAATGGAATGC-3’, and MF2: 5'-ATGGGAGTCAAACTTATCTTC-3’ and M2R-seq 5’-GCATCAACATCCAGAGCAAA-3’, respectively. The goal of using different reverse oligos was to generate shorter amplicons (~ 360 bp for *MlLuc1* and *MlLuc2*, and ~ 300 bp for *MlLuc3*), as Ion PGM sequencing requires PCR products of less than 400 bp. PCR conditions were as follows: initial denaturation at 95 C form 3 min, followed by 30 cycles comprising denaturation at 95 °C for 1 min, annealing at 50 C (*MlLuc1*) or 49 °C (*MlLuc2*) for 1 min and extension at 72 °C for 1 min, and a final extension step at 72 °C for 10 min. PCR products were resolved by agarose electrophoresis and isolated using the NucleoSpin Gel and PCR Clean-up kit (Macherey–Nagel).

#### Library preparation and sequencing

Barcoded libraries were prepared using the Ion Plus Fragment Library Kit (Life Technologies) and Ion Xpress Barcode Adapters 1–96 Kit (Life Technologies), following the manufacturer’s instructions (revision A.0 “Prepare Amplicon Libraries without Fragmentation Using the Ion Plus Fragment Library Kit”). The excess of non-ligated adapters was washed away with Agentcourt AMPure XP.

Template preparation and enrichment were carried out with the Ion One Touch 400 Template kit v2 DL (rev. 5.0) on the Ion One Touch System. Quality control of the resulting Ion Sphere Particles was performed with the Ion Sphere Quality Control Kit with the aid of a Qubit 2.0 fluorometer (Life Technologies).

The template-coated Ion Sphere particles were then deposited in an Ion-318 chip (Ion PGM 400 Sequencing Kit) and sequenced.

#### Data analysis

Ion PGM reads were processed with the Ion Torrent Suite software, which scored the quality of the runs and sorted the data according to the barcodes.

SAMtools (http://www.htslib.org/) was used to explore the alignments and calculate sequencing statistics (e.g., mean coverage depth per base, indel rate, etc.). Regions containing the priming sites were excluded from the analyses.

#### Genotyping and sequence verification

Alleles in each sample were called using *Amplian* (*amplian.py*), a complement of the ShoRAH package^30^ specifically designed for the amplicon mode. This program generates a multiple sequence alignment and assesses genetic variations by correcting sequencing errors, assembling reads, and estimating their frequencies. Following the authors’ recommendation, only sequences with a quality of reconstruction (posterior probability of the haplotype) > 0.95 were considered.

The sequences resulting from this filtering process underwent further manual scrutiny, with reads containing short indels (one or two nucleotides) being excluded. This step was necessary as indel formation is a frequent and strand-biased artefact of this sequencing technology^31^. Short nucleotide indels and stop codons were only permitted if they were also present in the cloned sequences.

Haplotypes represented by less than 15% of the reads from each individual were excluded, unless they were also detected by PCR-cloning and Sanger sequencing or found in multiple samples.

### Evolutionary analyses

A phylogeny based on the full-length haplotypes obtained by PCR-cloning and Sanger sequencing (Table 1) was inferred using the Neighbor-Joining method. The high level of divergence means that intron sequences of *MlLuc1* sequences cannot be unambiguously aligned with those from *MlLuc2* nor *MlLuc3*. Thus, only synonymous sites were used for this purpose. Evolutionary distances were computed using the modified Nei-Gojobori method (assumed transition/transversion bias = 2). The rate of variation among synonymous sites was modelled with a gamma distribution (shape parameter = 1), and the reliability of the tree topology was tested by bootstrapping (1000 replicates). These analyses were conducted using MEGA v11 package^32^.

Nucleotide variation within gene families was estimated separately at silent (synonymous and non-coding) and replacement (nonsynonymous) positions. Nucleotide diversity was quantified using Nei’s π^33^ with Jukes and Cantor correction^34^, π(JC). Genetic divergence, the average proportion of nucleotide differences between populations or species, was approximated using *K*(JC), average number of nucleotide substitutions per site between populations, or between species, with Jukes and Cantor correction^33^ (*K*_*A*_ and *K*_S_, at nonsynonymous and silent sites, respectively). These parameters, as well as the Tajima’s *D*^35^ and the haplotype diversity (*Hd*)^33^, were estimated with DnaSP v6^36^.

The McDonald & Kreitman test^37^ was used to test the hypothesis of neutral evolution. This test compares the rates of variation at silent and replacement positions within and between species. Under neutral expectations, the ratio of replacement to silent substitutions that are fixed between species should equal the ratio of replacement to silent variants that are polymorphic within species. Genomic sequences of *Luc1* and *Luc2* from *M. pacifica* and *M. okhotensis* were used for interspecific comparisons (AB371096.1, AB371097.1, AB674505.1, AB674506.1). Since no *Luc3* sequences were available for these species, *MlLuc2* sequences were used instead for interspecific comparisons of *MlLuc3*. Alignment gaps were excluded from these analyses. These calculations were carried out using DnaSP v6^36^.

Given that it was not possible to unambiguously determine the exon 1-intron 1 boundary for *MlLuc3* sequences (see the results section) and that exon 2 is only present in *MlLuc1* sequences, these regions were excluded from the evolutionary analyses, which encompassed from nucleotide position 459 to the 5’ end of the *M. pacifica Lucl* reference sequence (AB371096.1). The analysed segment represents 70% of the luciferase genes coding sequence and includes the full C-terminal catalytic domains 1 and 2 responsible for the luciferase activity^6,25^.

The sequence alignment and the phylogenetic analysis of the protein sequences were performed using Clustal Omega^38^. Sequences of Metridia species were aligned to identify conserved, semi-conserved, non-conserved, or identical regions.

### Cell culture and transfection

The HEK-293 cell line was maintained in low glucose Dulbecco’s modified Eagle’s medium (DMEM, Sigma-Aldrich) supplemented with 10% fetal bovine serum (FBS, Thermo Fisher). Cell cultures were maintained at 37 ◦C and 5% CO2. The pcDNA3.1(+) plasmid harboring a neomycin (G418) resistance was used for mammalian expression of the different *Metridia lucens* luciferases cDNAs (*MlLuc1*, *MlLuc2* and *MlLuc3*)^19^. Cells were grown to 60–70% confluence, the medium was replaced with DMEM 0% FBS, and the plasmids were transfected with 15 μg/ml of DNA:7.5 μg/ml of branched polyethylenimine (PEI 25, Sigma-Aldrich).

### Luciferase assays

HEK-293 cells were seeded in 24-well plates at 15,000 cells per well. Cells were subsequently transfected with pcDNA3.1(+) vectors carrying the different luciferase cDNAs and pCMV-β-Gal (Clontech, Mountain View, CA, USA). The data from three independent experiments were normalized using beta-galactosidase activity. To a solution containing 50 μL cell culture supernatant was added 50 μL of coelenterazine (2.4 nM) with mixing and incubated at RT in a small-volume cuvette. Spectra were collected using a wavelength-calibrated FluoroMax-3 fluorimeter (Horiba Jobin Yvon).

### Alphafold modelling of *Metridia lucens *luciferases

AlphaFold3 prediction and modelling were performed using the Galician Supercomputing Center (CESGA)^39^. The protein sequences accession numbers analysed were BAD93333 (*Metridia pacifica* luciferase), APQ47582 (*Metridia longa* luciferase 2), AJC98141 (*Metridia longa* luciferase 7). Molecular graphics and analyses were performed with UCSF ChimeraX^40^.

## Supplementary Information

Supplementary Information 1.

Supplementary Information 2.

Supplementary Information 3.

Supplementary Information 4.

Supplementary Information 5.

Supplementary Information 6.

Supplementary Information 7.

Supplementary Information 8.

Supplementary Information 9.

Supplementary Information 10.

## Data Availability

The sequences have been deposited in the Genbank under accession numbers PV925230, PV925230, PV925231, PV925232, PV925233, PV925234, PV925235, PV925236, PV925237, PV925238, PV925239, PV925240, PV925241, PV925242, PV925243, PV925244, PV925245, PV925246, PV925247, PV925248, PV925249, PV925250, PV925251, PV925252, PV925253, PV925254, PV925255, PV925256, PV925257, PV925258, PV925259, PV925260, PV925261, PV925262, PV925263, PV925264, PV925265, PV925266, PV925267, PV92568, PV925269, PV925270, PV925271, PV925272, PV925273, PV925274, PV925275, PV925276, PV925277, PV925278, PV925279, PV925280, PV925281, PV925282, PV925283, PV925284, PV925285, PV925286, PV925287, PV925288, PV925289, PV925290, PV925291, PV925292, PV925293, PV925294, PV925295, PV925296, PV925297, PV925298, PV925299, PV925300, PV925301, PV925302, PV925303, PV925304, PV925305, PV925306, PV925307, PV925308, PV925309, PV925310, PV925311, PV925312, PV925313, PV925314, PV925315, PV925316, PV925317, PV925318, PV925319, PV925320, PV925321, PV925322, (https://www.ncbi.nlm.nih.gov/genbank/).
