# Supplementary material for: Functional and evolutionary diversification of luciferase genes in Metridia lucens Boeck 1865
Source: Sci Rep. 2026 Jan 23;16:6032. doi: 10.1038/s41598-026-36319-2 (PMC12902078; doi:10.1038/s41598-026-36319-2)
Supplement: Supplementary file 2 — Supplementary Information 2. [file 41598_2026_36319_MOESM2_ESM.pdf]

Supplemental Table 2. Genetic diversity, π (JC) in %, at silent and nonsynonymous sites across *Luc* genes in *M. lucens*.

		Silent					Nonsynonymous				
Gene	*S*	*L*	*N*	π_S (JC)	*D*		*L*	*N*	π_A (JC)	*D*	
MlLuc1	16	297.1	27	16.0	-1.82	*	345.9	16	0.8	-1.81	*
MlLuc2	7	372.1	16	15.6	-0.69		344.9	1	0.1	-1.01	
MlLuc3	11	278.5	8	13.0	1.01		298.5	6	0.7	0.15	

S , number of sequences; *L* , number of sites; *N* , number of mutations, *D* , Tajima's *D* .
