# Supplementary material for: Functional and evolutionary diversification of luciferase genes in Metridia lucens Boeck 1865
Source: Sci Rep. 2026 Jan 23;16:6032. doi: 10.1038/s41598-026-36319-2 (PMC12902078; doi:10.1038/s41598-026-36319-2)
Supplement: Supplementary file 4 — Supplementary Information 4. [file 41598_2026_36319_MOESM4_ESM.pdf]

Supplemental Table 4. Haplotype diversity for *MlLuc1* from PCS (PCR-Cloning and Sanger sequencing) and MPS (Massive Parallel Sequencing) data.

Haplotypes		PCS data (No. of clones)							MPS data (No. of reads)		
MPS	PCS (corresp.)	Pool 1	Pool 2	Ind. 1	Ind. 2	Ind. 3	Ind. 8	Ind. 10	Ind. 8	Ind. 9	Ind. 10
MPS-M1.1	PCS-M1.1/M1.2	1	1	2			7	4	254	404	103
MPS-M1.2							1				
MPS-M1.3	PCS-M1.3/M1.4		1								
MPS-M1.4								1			
MPS-M1.5								1			
MPS-M1.6	PCS-M1.9		1								
MPS-M1.7								1			
MPS-M1.8	PCS-M1.6	1						1			44
MPS-M1.9									28		16
MPS-M1.10								1			
MPS-M1.11	PCS-M1.7		1								
MPS-M1.12	PCS-M1.8			1							
MPS-M1.13	PCS-M1.5	1									
MPS-M1.14									4		4
MPS-M1.15							1				
MPS-M1.16								1			
MPS-M1.17								1			
MPS-M1.18								1			
MPS-M1.19	PCS-M1.10	1									
MPS-M1.20	PCS-M1.11	1									
MPS-M1.21	PCS-M1.12		1								
MPS-M1.22	PCS-M1.13				1			3			26
MPS-M1.23	PCS-M1.14					1					
MPS-M1.24	PCS-M1.15	1									
MPS-M1.25							1	2	17	18	90
MPS-M1.26								1			
MPS-M1.27								1			
MPS-M1.28								1			
MPS-M1.29							2				
MPS-M1.30								1			
MPS-M1.31								1			

Haplotypes	Exon 1								Intron 1								Exon 2																					Intron 2													
MPS	**16**	**28**	**30**	**31**	62	81	**84**	**89**	100	109	113	127	132	137	138	144	188	205	219	229	**230**	232	235	**239**	**242**	**243**	**244**	**246**	**247**	**251**	**252**	**255**	**256**	**261**	262	**263**	271	283	284	286	296	298	300	302	312	346	358	359	360	361	362
MPS-M1.1	G	T	T	A	C	T	C	C	A	A	G	T	A	C	G	A	–	C	T	C	G	G	G	A	A	A	A	T	C	A	G	A	A	C	G	T	A	A	G	A	A	A	T	A	C	A	T	C	T	T	C
MPS-M1.2	.	.	.	.	.	.	.	.	.	.	.	.	.	.	.	.	–	.	.	.	.	.	.	.	.	.	.	.	.	.	.	.	.	.	.	.	T	.	.	.	.	.	.	.	.	.	.	.	.	.	.
MPS-M1.3	.	.	.	.	.	.	.	.	.	.	.	A	.	.	.	.	–	.	.	.	.	.	.	.	.	.	.	.	.	.	.	.	.	.	.	.	.	.	.	.	.	.	.	.	.	.	.	.	.	.	.
MPS-M1.4	.	**C**	**C**	.	.	.	.	.	.	.	.	.	.	.	.	.	–	.	.	.	.	.	.	.	.	.	.	.	.	.	.	.	.	.	.	.	.	.	.	.	.	.	.	.	.	.	.	.	.	.	.
MPS-M1.5	.	**C**	**C**	.	.	.	.	.	.	.	.	.	.	.	.	.	–	.	.	.	.	.	.	.	.	**T**	G	.	.	.	.	**G**	.	.	.	.	.	.	.	.	.	T	A	.	.	.	.	.	.	.	.
MPS-M1.6	.	**C**	**C**	.	.	.	.	.	.	.	.	.	.	.	.	.	–	.	.	.	**A**	.	.	.	.	.	**T**	.	.	.	.	**G**	.	.	.	.	.	.	.	.	.	.	.	.	.	.	.	.	.	.	.
MPS-M1.7	.	.	.	.	.	.	.	.	.	.	.	.	.	.	.	.	–	.	.	.	**A**	.	.	.	.	.	.	.	.	.	.	.	.	.	.	.	.	.	.	.	.	.	.	.	.	.	.	.	.	.	.
MPS-M1.8	.	.	.	.	.	.	.	.	.	.	.	.	.	.	.	.	–	.	.	.	.	.	.	.	.	.	**T**	**C**	.	.	.	**G**	.	.	.	.	.	.	.	.	.	.	.	.	.	.	.	.	.	.	.
MPS-M1.9	.	.	.	.	.	.	.	.	.	.	A	.	G	.	.	.	–	.	.	.	.	.	.	.	.	.	**T**	**C**	.	.	.	**G**	.	.	.	.	.	.	.	.	.	.	.	.	.	.	.	.	.	.	.
MPS-M1.10	.	.	.	.	.	.	.	.	.	.	.	.	.	T	.	C	–	.	.	.	.	.	.	.	.	.	**T**	**C**	.	.	.	**G**	.	.	.	.	.	.	.	.	.	.	.	.	.	.	.	.	.	.	.
MPS-M1.11	.	.	.	.	.	.	.	.	.	.	.	.	.	T	.	C	–	.	.	.	.	.	.	.	.	.	**T**	**C**	.	.	.	**G**	.	.	.	.	.	.	A	.	.	.	.	.	.	.	.	.	.	.	.
MPS-M1.12	.	.	.	.	.	.	**A**	.	.	.	.	.	.	.	.	.	A	.	.	.	.	.	.	.	.	.	**T**	**C**	.	.	.	.	.	.	.	.	.	.	.	.	.	.	.	G	.	.	.	.	.	.	.
MPS-M1.13	.	.	.	.	.	.	.	.	.	G	.	.	.	.	A	.	–	.	.	T	.	.	.	.	.	.	**T**	**C**	.	.	.	**G**	.	.	.	.	.	.	.	.	.	.	.	.	.	.	.	.	.	.	.
MPS-M1.14	.	.	.	.	.	.	.	.	.	.	.	.	.	.	A	.	–	.	.	.	.	.	.	**G**	.	.	**T**	**C**	.	.	.	**G**	.	.	A	**C**	.	.	.	.	.	.	.	.	.	.	.	.	.	.	.
MPS-M1.15	.	.	.	.	A	.	.	.	.	.	.	.	.	.	.	.	–	.	.	.	.	.	.	.	.	.	**T**	**C**	.	.	.	**G**	.	**T**	.	.	.	.	.	.	.	.	.	.	.	.	.	.	.	.	.
MPS-M1.16	.	.	.	**C**	.	.	.	.	.	.	.	.	.	.	.	.	–	.	.	.	.	.	A	.	.	.	**T**	**C**	.	.	**A**	**G**	.	.	.	.	.	.	.	.	.	.	.	.	.	.	.	.	.	.	.
MPS-M1.17	.	.	.	.	.	A	.	.	.	.	.	.	.	.	.	.	–	.	**A***	.	.	.	.	.	.	.	**T**	**C**	.	.	.	**G**	.	.	.	.	.	.	.	.	.	.	.	.	.	.	.	.	.	.	.
MPS-M1.18	.	.	.	.	.	.	.	.	.	.	.	.	.	.	.	.	–	.	.	.	.	.	A	.	.	.	**T**	**C**	.	.	.	**G**	.	.	.	.	.	.	.	T	.	.	.	.	.	.	.	.	.	.	.
MPS-M1.19	.	.	.	.	.	.	.	.	.	.	.	.	.	.	.	.	–	.	.	.	.	.	.	.	.	.	**T**	.	**G**	**G**	.	**G**	.	.	.	.	.	.	.	.	.	.	.	.	.	.	–	–	–	–	–
MPS-M1.20	.	.	.	.	.	.	.	.	.	.	.	.	.	.	.	.	–	.	.	.	.	.	.	.	.	.	**T**	.	.	**G**	.	**G**	.	.	.	.	.	.	.	.	C	.	.	.	.	.	.	.	.	.	.
MPS-M1.21	.	.	.	.	.	.	.	.	.	.	.	.	.	T	.	.	–	.	.	T	.	.	.	.	.	.	**T**	.	.	**G**	.	**G**	.	.	.	.	.	.	.	.	.	.	.	.	.	.	.	.	.	.	.
MPS-M1.22	.	.	.	.	.	.	.	.	.	.	.	.	.	T	.	.	–	.	.	.	.	.	.	.	.	.	**T**	.	.	**G**	.	**G**	.	.	.	.	.	.	.	.	.	.	.	.	.	.	.	.	.	.	.
MPS-M1.23	.	.	.	.	.	.	.	.	.	.	.	.	.	.	.	.	–	.	.	.	.	.	.	.	.	.	**T**	.	.	**G**	.	**G**	.	.	.	.	.	C	.	.	.	.	.	.	.	.	.	.	.	.	.
MPS-M1.24	**A**	.	.	.	.	.	.	.	G	.	.	.	.	.	.	.	–	.	.	T	.	.	.	.	.	.	**T**	.	.	**G**	.	**G**	**T**	.	.	.	.	.	.	.	.	.	.	.	.	.	.	.	.	.	.
MPS-M1.25	.	.	.	.	.	.	.	.	.	.	.	.	.	.	.	.	–	.	.	.	.	.	.	.	.	.	**T**	.	.	**G**	.	**G**	.	.	.	.	.	.	.	.	.	.	.	.	.	.	.	.	.	.	.
MPS-M1.26	.	.	.	.	.	.	.	**T**	.	.	.	.	.	.	.	.	–	.	.	T	.	.	.	.	.	.	**T**	.	.	**G**	.	**G**	.	.	.	.	.	.	.	.	.	.	.	.	.	.	.	.	.	.	.
MPS-M1.27	.	.	.	.	.	.	.	**T**	.	.	.	.	.	.	.	.	–	.	.	.	.	.	.	.	.	.	**T**	.	.	**G**	.	**G**	.	.	.	.	.	.	.	.	.	.	.	.	.	.	.	.	.	.	.
MPS-M1.28	.	.	.	.	.	.	.	.	.	.	.	.	.	.	.	.	–	.	.	.	.	.	.	.	.	.	**T**	.	.	**G**	.	**G**	.	.	.	.	.	.	.	.	.	.	.	.	.	–	.	.	.	.	.
MPS-M1.29	.	.	.	.	.	.	.	.	.	.	.	.	.	.	.	.	–	T	.	T	.	T	.	.	.	.	**T**	.	.	**G**	.	**G**	.	.	.	.	.	.	.	.	.	.	.	.	.	.	.	.	.	.	.
MPS-M1.30	.	.	.	.	.	.	.	.	.	.	.	.	.	.	.	.	–	.	.	.	.	.	.	.	**T**	.	**T**	.	.	**G**	.	**G**	.	.	.	.	.	.	.	.	.	.	.	.	A	.	.	.	.	.	.
MPS-M1.31	.	.	.	.	.	.	.	.	.	.	.	.	.	.	.	.	–	.	.	.	.	.	.	.	.	.	**T**	.	.	**G**	.	**C**	.	.	.	.	.	.	.	.	.	.	.	.	.	.	.	.	.	.	.

Note: Plain text, silent or intron mutation; Bold, nonsynonymous mutation; Red, nonsense/frameshift mutation; *, nonsense mutation (stop codon); Ligh grey, exon; dot, same as first sequence; –, deletion.
