# Supplementary material for: Functional and evolutionary diversification of luciferase genes in Metridia lucens Boeck 1865
Source: Sci Rep. 2026 Jan 23;16:6032. doi: 10.1038/s41598-026-36319-2 (PMC12902078; doi:10.1038/s41598-026-36319-2)
Supplement: Supplementary file 5 — Supplementary Information 5. [file 41598_2026_36319_MOESM5_ESM.pdf]

Supplemental Table 5. *MlLuc2* haplotypes obtained by means of PCS (PCR-cloning and Sanger sequencing) and MPS (Massive Parallel Sequencing) .

Haplotypes		PCS data (No. Clones)									MPS data (No. Reads			Exon 1				
MPS	PCS (corresp.)	Pool 1	Pool 2	Ind. 1	Ind. 2	Ind. 3	Ind. 4	Ind. 5	Ind. 6	Ind. 7	Ind. 8	Ind. 9	Ind. 10	**16**	21	**34**	39	64
MPS-M2.1	PCS-M2.1	1												A	C	G	C	G
MPS-M2.2	PCS-M2.3	1												·	·	·	·	·
MPS-M2.3	PCS-M2.2									1				·	·	·	·	·
MPS-M2.4	PCS-M2.4						1							**C**	·	·	·	T*
MPS-M2.5	PCS-M2.5				1									**C**	·	·	·	·
MPS-M2.6	PCS-M2.6				1							7		·	·	·	·	·
MPS-M2.7	PCS-M2.7				1								22	·	·	·	·	·
MPS-M2.8	PCS-M2.8			1					1		156			·	·	·	·	·
MPS-M2.9											168	2	135	·	·	·	·	·
MPS-M2.10											45	26	96	·	·	·	·	·
MPS-M2.11												24	6	·	·	·	·	·
MPS-M2.12	PCS-M2.9					1								·	T	**A**	A	·

Haplotypes	Intron 1																																
MPS	104	108	120	124	132	134	136	142	151	157	159	168	169	170	173	174	184	185	186	195	196	210	212	222	230	238	247	256	257	259	262	266	267
MPS-M2.1	G	G	G	C	G	G	A	A	–	G	C	G	C	C	A	G	–	–	A	T	G	G	T	T	G	A	A	T	C	A	C	T	T
MPS-M2.2	·	·	·	·	·	·	·	·	–	·	·	·	·	·	·	·	–	–	·	·	·	·	·	·	·	·	·	·	·	·	·	·	·
MPS-M2.3	·	·	·	A	·	·	G	–	T	A	T	·	·	·	·	·	–	–	·	·	·	A	·	·	·	·	G	·	T	·	·	·	A
MPS-M2.4	·	·	·	A	A	·	·	–	T	·	·	·	·	·	·	·	–	–	·	·	·	·	·	·	·	·	·	·	·	·	·	·	·
MPS-M2.5	·	·	·	A	A	·	·	–	T	·	·	·	·	·	·	·	–	–	·	·	·	·	·	·	·	·	·	·	·	·	·	·	·
MPS-M2.6	·	·	·	A	·	·	·	–	T	·	·	·	·	·	·	·	–	–	·	·	·	·	·	·	·	C	·	·	·	·	·	·	·
MPS-M2.7	·	·	·	A	·	·	·	–	T	·	·	·	·	·	·	·	–	–	·	·	·	·	·	·	·	·	·	·	·	·	·	·	·
MPS-M2.8	·	·	·	A	·	·	·	–	T	·	·	·	·	·	·	·	T	T	T	·	·	·	A	·	·	·	·	·	·	·	·	·	·
MPS-M2.9	·	·	·	A	·	·	·	–	–	·	·	·	·	·	·	·	–	–	·	·	·	·	A	·	·	·	·	·	·	·	·	·	·
MPS-M2.10	·	·	·	A	·	·	·	–	–	·	·	·	·	·	·	·	–	–	·	·	·	·	·	·	·	·	·	·	·	·	·	·	·
MPS-M2.11	·	·	C	A	·	·	·	–	–	·	·	·	·	·	·	C	–	–	·	·	·	·	·	·	·	·	·	·	·	·	·	·	·
MPS-M2.12	A	T	·	A	C	T	·	–	–	·	·	T	G	T	T	·	–	–	·	G	A	·	·	A	T	·	·	C	·	T	T	G	·

Haplotypes	Intron 1																											Exon 2	
MPS	272	274	275	276	277	278	279	280	281	282	283	284	285	286	287	295	303	304	306	310	322	323	325	326	331	332	333	342	**359**
MPS-M2.1	A	A	A	A	A	A	T	T	G	A	A	A	A	C	G	T	G	T	C	C	G	A	A	T	T	T	A	T	A
MPS-M2.2	·	·	·	·	·	·	·	·	·	·	·	·	·	·	·	·	·	·	·	·	·	·	·	·	·	·	·	·	**G**
MPS-M2.3	·	·	·	·	·	·	·	·	·	·	·	·	·	A	·	C	·	·	·	T	C	C	·	·	·	·	·	C	·
MPS-M2.4	·	·	·	·	·	·	·	·	·	·	·	·	·	·	·	·	·	·	·	·	C	·	·	·	·	·	·	C	·
MPS-M2.5	·	·	·	·	·	·	·	·	·	·	·	·	·	·	·	·	·	·	·	·	C	·	·	·	·	·	·	C	·
MPS-M2.6	·	·	·	·	·	·	·	·	·	·	·	·	·	T	A	·	·	·	·	·	C	·	·	A	·	·	·	C	·
MPS-M2.7	·	C	·	·	·	·	·	·	·	·	·	·	·	·	·	·	·	·	·	·	C	·	·	·	·	·	·	C	·
MPS-M2.8	·	C	·	·	·	·	·	·	·	·	·	·	·	·	·	·	·	·	·	·	C	·	·	·	C	C	C	C	·
MPS-M2.9	·	C	·	·	·	·	·	·	·	·	·	·	·	·	·	·	·	·	·	·	C	·	·	·	·	·	·	C	·
MPS-M2.10	·	·	·	·	·	·	·	·	·	·	·	·	·	·	·	·	·	·	·	·	C	·	·	·	·	·	·	C	·
MPS-M2.11	·	·	·	·	·	·	·	·	·	·	·	·	·	·	·	·	·	·	·	·	C	·	·	·	·	·	·	C	·
MPS-M2.12	·	·	–	–	–	–	–	–	–	–	–	–	–	·	·	·	A	A	G	·	C	·	G	·	·	A	·	C	·

Note: Plain text, silent or intron mutation; Bold, nonsynonymous mutation; Red, nonsense */frameshift mutation; Ligh grey, exon; dot, same as first sequence; –, deletion.
