# Supplementary material for: Functional and evolutionary diversification of luciferase genes in Metridia lucens Boeck 1865
Source: Sci Rep. 2026 Jan 23;16:6032. doi: 10.1038/s41598-026-36319-2 (PMC12902078; doi:10.1038/s41598-026-36319-2)
Supplement: Supplementary file 6 — Supplementary Information 6. [file 41598_2026_36319_MOESM6_ESM.pdf]

Supplemental Table 6. Haplotype diversity at the *MlLuc3* locus from PCS (PCR-Cloning and Sanger sequencing) and MPS (Massive Parallel Sequencing) data.

| Haplotypes | | PCS data (No. Clones) | | | | | | | | | MPS data (No. Reads) | | |
|---|---|---|---|---|---|---|---|---|---|---|---|---|---|
| MPS | PCS (corresp.) | Pool 1 | Pool 2 | Ind. 1 | Ind. 2 | Ind. 3 | Ind. 4 | Ind. 5 | Ind. 6 | Ind. 7 | Ind. 8 | Ind. 9 | Ind. 10 |
| MPS-M3.1 | PCS-M3.1, PCS-M3.2, PCS-M3.13 | | 1 | | 1 | | 1 | | 3 | 2 | 1248 | 1361 | 112 |
| MPS-M3.2 | PCS-M3.14 | | 1 | | | | | | | | | | |
| MPS-M3.3 | PCS-M3.11 | 1 | | | | | | | | | | | |
| MPS-M3.4 | | | | | | | | | | | 41 | 75 | 60 |
| MPS-M3.5 | PCS-M3.10 | | | | | | | | | 1 | | | |
| MPS-M3.6 | PCS-M3.15 | | | 1 | | | | | | | | | |
| MPS-M3.7 | PCS-M3.5, PCS-M3-6, PCS -M3.7 | 1 | | | | | | 3 | | | | | 3 |
| MPS-M3.8 | | | | | | | | | | | 56 | 83 | 19 |
| MPS-M3.9 | PCS-M3.3, PCS-M3.4, PCS-M3.8 | 1 | | | | | | 4 | | | | | |
| MPS-M3.10 | PCS-M3.9 | | | | | | | 1 | | | | | |
| MPS-M3.11 | PCS-M3.12 | | 1 | | | | | | | | | | |

| MPS | 21 | 26 | 28 | 30 | 31 | 33 | 48 | 52 | 58 | 61 | 70 | 74 | 75 | 76 | 77 | 78 | 79 | 80 | 81 | 82 | 83 | 84 | 85 | 86 | 87 | 88 | 89 | 90 | 91 | 92 | 93 | 110 | 147 | 162 | 178 | 182 | 223 | 242 | 254 | 274 |
|---|---|---|---|---|---|---|---|---|---|---|---|---|---|---|---|---|---|---|---|---|---|---|---|---|---|---|---|---|---|---|---|---|---|---|---|---|---|---|---|---|
| MPS-M3.1 | T | T | C | T | T | T | G | C | A | T | C | T | T | T | T | T | G | T | T | – | – | A | C | A | A | A | G | A | C | T | T | T | C | T | A | A | A | C | A | C |
| MPS-M3.2 | C | . | G | . | . | . | A | G | . | A | . | . | – | – | – | – | – | – | – | – | – | – | – | – | – | – | – | – | – | – | – | . | . | . | . | . | . | . | . | . |
| MPS-M3.3 | . | . | . | . | . | . | . | G | . | A | . | . | . | . | . | . | T | . | . | T | T | . | . | . | . | . | . | . | A | . | . | . | . | . | . | . | . | . | . | A |
| MPS-M3.4 | . | . | . | . | . | . | . | . | . | A | . | . | . | . | . | . | . | . | . | – | – | . | . | . | . | . | . | . | . | . | . | . | . | . | G | . | . | . | . | . |
| MPS-M3.5 | . | . | . | . | . | . | . | . | . | A | . | C | . | . | . | . | . | . | . | – | – | . | . | . | . | . | . | . | . | . | . | . | . | . | G | . | . | . | . | . |
| MPS-M3.6 | . | . | . | . | . | . | . | . | . | A | A | . | . | . | . | . | . | . | . | – | – | . | . | . | . | . | . | . | . | . | . | . | . | . | . | . | . | . | . | . |
| MPS-M3.7 | . | . | . | . | . | . | . | . | G | . | . | . | . | . | . | . | . | . | . | – | – | . | . | . | . | . | . | . | . | . | . | . | . | G | . | G | G | . | . | . |
| MPS-M3.8 | . | . | . | . | . | . | . | . | . | . | . | . | . | . | . | . | . | . | . | – | – | . | . | . | . | . | . | . | . | . | . | . | A | . | . | . | . | . | C | . |
| MPS-M3.9 | . | . | . | . | . | . | . | . | . | . | . | . | . | . | . | . | . | . | . | – | – | . | . | . | . | . | . | . | . | . | . | A | A | . | . | . | . | . | C | . |
| MPS-M3.10 | . | . | . | . | . | . | . | . | . | . | . | . | . | . | . | . | . | . | . | – | – | . | . | . | . | . | . | . | . | . | . | . | . | . | . | . | . | A | . | . |
| MPS-M3.11 | . | C | . | A | A | A | . | . | . | . | . | . | . | . | . | . | . | . | . | – | – | . | . | . | . | . | . | . | . | . | . | . | . | . | . | . | . | . | . | . |

Note: Plain text, silent or intron mutation; Bold, nonsynonymous mutation; Red, nonsense/frameshift mutation; *, nonsense mutation (stop codon); Ligh grey, exon; dot, same as first sequence; –, deletion.
Intron and exon boundaries for this region were difficult to determine, so all positions were considered as noncoding
