# Supplementary material for: Functional and evolutionary diversification of luciferase genes in Metridia lucens Boeck 1865
Source: Sci Rep. 2026 Jan 23;16:6032. doi: 10.1038/s41598-026-36319-2 (PMC12902078; doi:10.1038/s41598-026-36319-2)
Supplement: Supplementary file 7 — Supplementary Information 7. [file 41598_2026_36319_MOESM7_ESM.pdf]

Supplemental Table 7. Estimates (in %) of nucleotide divergence at synonymous (K_S) and nonsynonymous (KA) sites (below and above the diagonal) across Luc genes and species.

	MlLuc1	*MpLuc1*	*MoLuc1*	*MlLuc2*	*MpLuc2*	*MoLuc2*	*MlLuc3*
MlLuc1	-	1.1	3.0	18.2	18.5	18.7	23.5
MpLuc1	11.6	-	2.5	18.5	18.2	19.0	23.9
MoLuc1	25.4	26.1	-	19.3	18.8	19.6	24.6
MlLuc2	78.4	96.0	84.9	-	2.85	3.3	3.5
MpLuc2	83.0	100.4	78.0	7.8	-	3.0	2.9
MoLuc2	78.5	86.1	67.3	18.1	19.2	-	2.7
MlLuc3	84.1	94.5	81.3	16.4	18.8	14.9	-
