# Supplementary material for: Functional and evolutionary diversification of luciferase genes in Metridia lucens Boeck 1865
Source: Sci Rep. 2026 Jan 23;16:6032. doi: 10.1038/s41598-026-36319-2 (PMC12902078; doi:10.1038/s41598-026-36319-2)
Supplement: Supplementary file 8 — Supplementary Information 8. [file 41598_2026_36319_MOESM8_ESM.pdf]

Supplemental Table 8. McDonald and Kreitman's test.

Outgroup	*MlLuc1*		*MlLuc2*		*MlLuc3*	
M. pacifica	Fixed	Polym	Fixed	Polym	Fixed	Polym
Silent	38	27	22	13	28	5
Replacement	1	16	8	1	6	6
p	0.000		0.233		0.044	
M. okhotensis	Fixed	Polym	Fixed	Polym	Fixed	Polym
Silent	67	27	43	16	35	7
Replacement	8	16	9	1	7	6
p	0.002		0.431		0.057	
