# Supplementary figures and images for: Functional and evolutionary diversification of luciferase genes in Metridia lucens Boeck 1865
Source: Sci Rep. 2026 Jan 23;16:6032. doi: 10.1038/s41598-026-36319-2 (PMC12902078; doi:10.1038/s41598-026-36319-2)

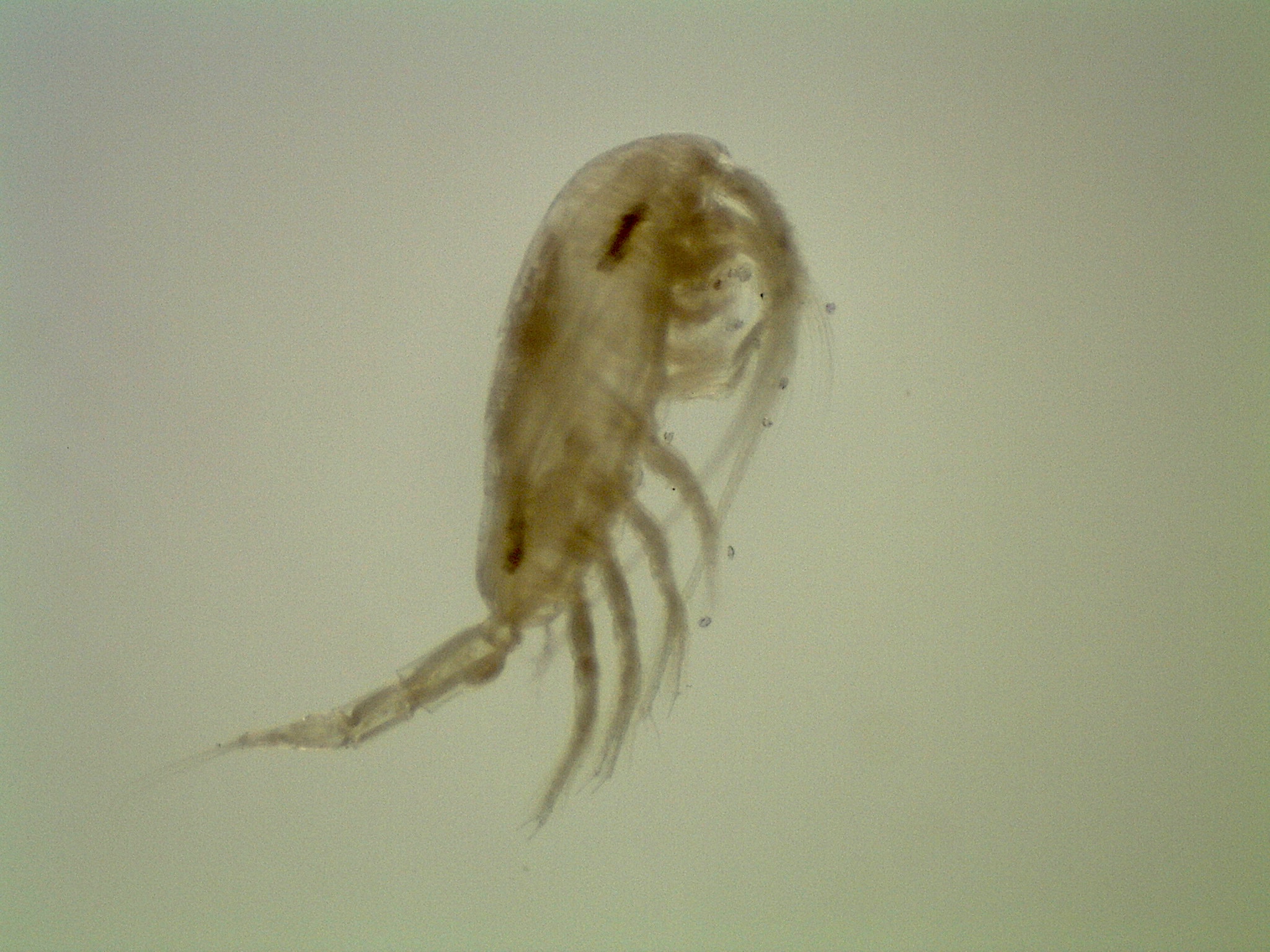

Supplement: Supplementary file 9 — Supplementary Information 9. [file 41598_2026_36319_MOESM9_ESM.jpg]
