# Supplementary material for: Functional and evolutionary diversification of luciferase genes in Metridia lucens Boeck 1865
Source: Sci Rep. 2026 Jan 23;16:6032. doi: 10.1038/s41598-026-36319-2 (PMC12902078; doi:10.1038/s41598-026-36319-2)
Supplement: Supplementary file 10 — Supplementary Information 10. [file 41598_2026_36319_MOESM10_ESM.pdf]

Motif 1

```
#PCS-M1.1                                  ANSDADR--- GKMPGKKLPL EVLIEMEANA RKAGCTRGCL ICLSKIKCTA KMKVYIPGRC HDYGGDKKTG QGGIVGA--I VDIPEISGFK
#PCS-M1.2                                  .......--- .......... .......... .......... .......... .......... .N........ .......--. ..........
#PCS-M1.3                                  .......--- .......... .......... .......... .......... .......... .N........ .......--. ..........
#PCS-M1.4                                  .......--- .......... .......... .......... ...*...... .......... N......... .......--. ..........
#PCS-M1.5                                  .......--- .......... .......... .......... .......... .......... .......... .......--. ..........
#PCS-M1.6                                  .......--- .......... .......... .......... M......... .......... .......... .......--. ..........
#PCS-M1.7                                  .......--- .......... .......... .......... .......... .......... ........S. .......--. ..........
#PCS-M1.8                                  .......--- .......... .......... H......... M......... .......... .......... .......--. ..........
#PCS-M1.9                                  .......--- .......... .......... .......... .......... .......... ........S. .......--. ..........
#PCS-M1.10                                 .......--- .......... .......... .......... .......... .......... .......... .A.....--. ..........
#PCS-M1.11                                 ....G..--- .......... .......... .......... .......... .......... .......... .A.....--. ..........
#PCS-M1.12                                 .......--- .......... .......... .......... .......... .......... .......... .A.....--. ..........
#PCS-M1.13                                 .......--- .......... .......... .......... .......... .......... .......... .A.....--. ..........
#PCS-M1.14                                 .......--- .......... .......... .......... .......... .......... .......... .A.....--. ..........
#PCS-M1.15                                 ......*--- .......... ...LK..... .......... .......... .......... .......... ...V...--. ..........
#MpLuc1_M._pacifica_(AB371096.1)           V......--- .......... .......... .......... .......... .......... .......... .A.....--. ..........
#MoLuc1_M._okhotensis_(AB674505.1)         ..A....--- .......... A......... F......... .......... ...E...... .......... .A.....--. ..........
#MLuc2-1                                   ---..N.GGH .GH....M.K ...L...... KR...A.... ....H..... ...KF..... .S.A...DSA ....-SDEET ..M...P...
#PCS-M2.1                                  ---..N.GGH .GH....M.K ...L...... KR...A.... ....H..... ...KF..... .S.A...DSA ....-SDEET ..M...P...
#PCS-M2.2                                  ---..N.GGH .GH....M.K ...L...... KR...A.... ....H..... ...KF..... .S.A...DSA ....-SDEET ..M...P...
#PCS-M2.3                                  ---G.N.--- .GH....M.K ...L...... KR...A.... ....H..... ...KF..... .S.A...DSA ....-SDEET ..M...P...
#PCS-M2.4                                  ---..N.GGH .GH....M.K ...L...... KR...A.... ....H..... ...KF..... .S.A...DSA ....-SDEET ..M...P...
#PCS-M2.5                                  ---..N.GGH .GH....M.K ...L...... KR...A.... ....H..... ...KF..... .S.A...DSA ....-SDEET ..M...P...
#PCS-M2.6                                  ---..N.GGH .GH....M.K ...L...... KR...A.... ....H..... ...KF..... .S.A...DSA ....-SDEET ..M...P...
#PCS-M2.7                                  ---..N.GGH .GH....M.K ...L...... KR...A.... ....H..... ...KF..... .S.A...DSA ....-SDEET ..M...P...
#PCS-M2.8.1_Recomb                         ---..N.GGH .GH....M.K ...L...T.. KR...H.... V...H....K ...KF..... .S.A...DSA ....-TEEET ..M...P...
#PCS-M2.9_Recomb                           ---..N.GGH .GH....M.K ...L...T.. KR...H.... V...H....K ...KF..... .S.A...DSA ....-TEEET ..M...P...
#MpLuc2_M._pacifica_(AB371097.1)           ---..N.GGH .GH....M.K ...V...... KR...H.... ....H....K ...KF..... .S.E...DSA ....--GEE. ..M...P...
#MoLuc2_M._okhotensis_(AB674506.1)         ---..N.GGH .GH....M.K ...L...... KR...H.... ....H....Q ...KF..... .S.A...DSA ....-TEEET ..M...A...
#PCS-M3.1_Recomb                           ---..N.GGH .GH....M.K ...L...T.. KR...H.... V...H....K ...KF..... .S.A...DSA ....-TDEET ..M...P...
#PCS-M3.8                                  ---..N.GGH .GH....M.K ...L...T.. KR...H.... V...H....K ...KF..... .S.A...DSA ....-TEEET ..M...P...
#PCS-M3.2_Recomb                           ---..N.GGH .GH....M.K ...L...T.. KR...H.... V...H....K ...KF..... .S.A...DSA ....-TDEET ..M...P...
#PCS-M3.11_Recomb                          ---..N.GGH .GH....M.K ...L...... KR...H.... V...H....K ...KF..... .S.A...DSA ....-TEEET ..M...P...
#PCS-M3.3                                  ---..N.GGH .GH....M.K ...L...T.. KR...H.... V...H....K ...KF..... .S.A...DSA ....-TEEET ..M...P...
#PCS-M3.12                                 ---..N.--- .GH....M.K ...L...T.. KR...H.... V...H....K ...KF..... .S.A...DSA ....-TEEET ..M...P...
#PCS-M3.13                                 ---..N.--- .GH....M.K ...L...T.. KR...H.... V...H....K ...KF..... .S.A...DSA ....-TEEET ..M...P...
#PCS-M3.14_Recomb                          ---..N.GGH .GH....M.K ...L...T.. KR...H.... V...H....K ...KF..... .S.A...DSA ....-TEEET ..M...P...
#PCS-M3.4                                  ---..N.GGH .GH....M.K ...L...T.. KR...H.... V...H....K ...KF..... .S.A...DSA ....-TEEET ..M...P...
#PCS-M3.5                                  ---..N.GGH .GH....M.K ...L...T.. KR...H.... V...H....K ...KF..... .S.A...DSA ....-TEEET ..M...PV..
#PCS-M3.6                                  ---..N.GGH .GH....M.K ...L...T.. KR...H.... V...H....K ...KF..... .S.A...DSA ....-TEEET ..M...P...
#PCS-M3.7                                  ---..N.GGH .GH....M.K ...L...T.. KR...H.... V...H....K ...KF..... .S.A...DSA ....-TEEET ..M...P...
#PCS-M3.9_Recomb                           ---..N.GGH .GH....M.K ...L...T.. KR...H.... V...H....K ...KF..... .S.A...DSA ....-TDEET ..M...P...
#PCS-M3.10                                 ---..N.GGH .GH....M.K ...L...T.. KR...H.... V...H....K ...KF..... .S.A...DSA ....-TEEET ..M...P...
#PCS-M3.15                                 ---..N.GGH .GH....M.K ...L...T.. KR...H.... V...H....K ...KF..... .S.A...DSA ....-TEEET ..M...P...
#LoLuc1-3_Lucicutia_ovaliformis_(AB716356.1) ---.MSK-QH .--AAL...P D..D...C.. K.S..V.... Q..AL..... ..RK...... .S.E...DIA ....-.KELT I.....P..L
```

Motif 2

```
#PCS-M1.1                                 ELGPMEQFIA QVDLCADCTT GCLKGLANVK CSELLKKWLP DRCASFADKI QSEVDNIKGL ---------- ------A-G- --D---R*--
#PCS-M1.2                                 .......... .......... .......... .......... .......... .......... ---------- ------.-.- --.---..--
#PCS-M1.3                                 ......K... .......... .......... .......... .......... .......... ---------- ------.-.- --.---..--
#PCS-M1.4                                 .......... .......... .......... .......... .......... .......... ---------- ------.-.- --.---..--
#PCS-M1.5                                 .......... .......... .......... .......... .......... .......... ---------- ------.-.- --.---..--
#PCS-M1.6                                 .......... .G........ .......... .......... .......... .......... ---------- ------.-.- --.---..--
#PCS-M1.7                                 .......... .......... .......... .......... .......... .......... ---------- ------.-.- --.---..--
#PCS-M1.8                                 .......... .......... .......... .......... .......... .......... ---------- ------.-.- --.---..--
#PCS-M1.9                                 .......... .....T.... .......... .......... .......... .......... ---------- ------.-.- --.---..--
#PCS-M1.10                                .......... .......... .......... .......... ....G..... .......... ---------- ------.-.- --.---..--
#PCS-M1.11                                .......... .......... .......... .......... .......... .......... ---------- ------.-.- --.---..--
#PCS-M1.12                                .......... .......... .......... .......... .......... .......... ---------- ------.-.- --.---..--
#PCS-M1.13                                .......... .......... .......... .......... .......... .......... ---------- ------.-.- --.---..--
#PCS-M1.14                                .......... .......... .......... .......... .......... .......... ---------- ------.-.- --.---..--
#PCS-M1.15                                .......... .......... .......... .......... ....N..... .......... ---------- ------.-.- --.---..--
#MpLuc1_M._pacifica_(AB371096.1)          .......... .......... .......... ..A....... .......... .......... ---------- ------.-.- --.---..--
#MoLuc1_M._okhotensis_(AB674505.1)        .......... .......... .......... ..A....... .......... ....H..... ---------- ------.-.- --.---..--
#MLuc2-1                                  DKE..D.... .....V.... .........H ..D....... S..KT..S.. ..Q..T.... ---------- ------.-.- --.---.---
#PCS-M2.1                                 DKE..D.... .....V.... .........H ..D....... S..KT..S.. ..Q..T.... ---------- ------.-.- --.---..--
#PCS-M2.2                                 DKE..D.... .....V.... .........H ..D....... S..KT..S.. ..Q..T.... ---------- ------.-.- --.---..--
#PCS-M2.3                                 DKE..D.... .....V.... .........H ..D....... S..KT..S.. ..Q..T.... ---------- ------.-.- --.---..--
#PCS-M2.4                                 DKE..D.... .....V.... .........H ..D....... S..KT..S.. ..Q..T.... ---------- ------.-.- --.---..--
#PCS-M2.5                                 DKE..D.... .....V.... .........H ..D....... S..KT..S.. ..Q..T.... ---------- ------.-.- --.---..--
#PCS-M2.6                                 DKE..D.... .....V.... .........H ..D....... S..KT..S.. ..Q..T.... ---------- ------.-.- --.---..--
#PCS-M2.7                                 DKE..D.... .....V.... .........H ..D....... S..KT..S.. ..Q..T.... ---------- ------.-.- --.---..--
#PCS-M2.8.1_Recomb                        DKE..D.... .....V.... .........H ..D....... S..KT..S.. ..Q..T.... ---------- ------.-.- --.---..--
#PCS-M2.9_Recomb                          DKE..D..V. .....V.... .........H ..D..E.... S..KT..S.. P.Q..T.... ---------- ------.-.- --.---..--
#MpLuc2_M._pacifica_(AB371097.1)          DKE..D.... .....V.... .........H ..A....... S..KT..S.. ..Q..T.... ---------- ------.-.- --.---..--
#MoLuc2_M._okhotensis_(AB674506.1)        D.E....... .....V.... .........H ..D....... S..KT..S.. ..Q..T.... ---------- ------.-.- --.---..--
#PCS-M3.1_Recomb                          DKE..D.... .....V.... .........H ..D....... S..KT..S.. ..Q..T.... ---------- ------.-.- --.---..--
#PCS-M3.8                                 DKE..D.... .....V.... .........H ..D..E.... S..KT..S.. ..Q..T.... ---------- ------.-.- --.---..--
#PCS-M3.2_Recomb                          DKE..D.... .....V.F.. .........H ..D....... S..KT..S.. ..Q..T.... ---------- ------.-.- --.---..--
#PCS-M3.11_Recomb                         DKE..D...- ---------- ---------- ..D....... S..KT..S.. ..Q..T.... ---------- ------.-.- --.---..--
#PCS-M3.3                                 DKE..D...- ---------- ---------- ..D....... S..KT..S.. ..Q..T.... ---------- ------.-.- --.---G.--
#PCS-M3.12                                DKE..D...- ---------- ---------- ..D....... S..KT..S*. ..Q..T.... ---------- ------.-.- --.---..--
#PCS-M3.13                                DKE..D...- ---------- ---------- ..D....... S..KT..S.. ..Q..T.... ---------- ------.-.- --.---G.--
#PCS-M3.14_Recomb                         DKE..D...- ---------- ---------- ..D....... S..KT..S.. ..Q..T.... ---------- ------.-.- --.---G.--
#PCS-M3.4                                 DKE..D...- ---------- ---------- ..D....... S..KT..S.. ..Q..T.... ---------- ------.-.- --.---G.--
#PCS-M3.5                                 DKE..D.... .....V.... .........H ..D..E.... S..KT..S.. ..Q..T.... ---------- ------.-.- --.---..--
#PCS-M3.6                                 DKE..D...S .....V.... .........H ..D..E.... S..KT..S.. ..Q..T.... ---------- ------.-.- --.---..--
#PCS-M3.7                                 DKE..D.... .....V.... .........H ..D..E.... S..KT..S.. ..Q..T.... ---------- ------.-.- --.---..--
#PCS-M3.9_Recomb                          DKE..D.... .....V.... .........H ..D....... S..KT..S.. ..Q..T.... ---------- ------.-.- --.---..--
#PCS-M3.10                                DKE..D.... .....V.... .........H ..D..E.... S..KT..S.. ..Q..T.... ---------- ------.-.- --.---G.--
#PCS-M3.15                                DKE..D.... .....V.... .........H ..D....... S..KT..S.. ..Q..T.... ---------- ------.-.- --.---..--
#LoLuc1-3_Lucicutia_ovaliformis_(AB716356.1) D.A..D..V. .....V..SS R........Q ..CK.Y.... T..TG.QA.. KK.A.TVI.. EDALALGFDT IQACVA.-.K CK.TVG.YS*
```

Suplemental Figure 2. Aminoacid sequence alignment of Metridia luciferase genes. The two tandem functional motifs are highlighted in yellow.
